# An Alternative Method to Calculate Simplified Projected Aortic Valve Area at Normal Flow Rate

**DOI:** 10.5935/abc.20180018

**Published:** 2018-02

**Authors:** Joana Sofia Silva Moura Ferreira, Nádia Moreira, Rita Ferreira, Sofia Mendes, Rui Martins, Maria João Ferreira, Mariano Pego

**Affiliations:** Centro Hospitalar e Universitário de Coimbra, Serviço de Cardiologia, Coimbra - Portugal

**Keywords:** Aortic Valve Stenosis / diagnosis, Aortic Valve Stenosis / diagnostic imaging, Echocardiography, Stress, Heart Valves / physiopathology

## Abstract

**Background:**

Simplified projected aortic valve area (EOA_proj_) is a valuable echocardiographic parameter in the evaluation of low flow low gradient aortic stenosis (LFLG AS). Its widespread use in clinical practice is hampered by the laborious process of flow rate (Q) calculation.

**Objetive:**

This study proposes a less burdensome, alternative method of Q calculation to be incorporated in the original formula of EOA_proj_ and measures the agreement between the new proposed method of EOA_proj_ calculation and the original one.

**Methods:**

Retrospective observational single-institution study that included all consecutive patients with classic LFLG AS that showed a Q variation with dobutamine infusion ≥ |15|% by both calculation methods.

**Results:**

Twenty-two consecutive patients with classical LFLG AS who underwent dobutamine stress echocardiography were included. Nine patients showed a Q variation with dobutamine infusion calculated by both classical and alternative methods ≥ |15|% and were selected for further statistical analysis. Using the Bland-Altman method to assess agreement we found a systematic bias of 0,037 cm^2^ (95% CI 0,004 - 0,066), meaning that on average the new method overestimates the EOA_proj_ in 0,037 cm^2^ compared to the original method. The 95% limits of agreement are narrow (from -0,04 cm^2^ to 0,12 cm^2^), meaning that for 95% of individuals, EOA_proj_ calculated by the new method would be between 0,04 cm^2^ less to 0,12 cm^2^ more than the EOA_proj_ calculated by the original equation.

**Conclusion:**

The bias and 95% limits of agreement of the new method are narrow and not clinically relevant, supporting the potential interchangeability of the two methods of EOA_proj_ calculation. As the new method requires less additional measurements, it would be easier to implement in clinical practice, promoting an increase in the use of EOA_proj_.

## Introduction

Classical low-flow, low-gradient (LFLG) aortic stenosis (AS) is characterized by the combination of a calcified aortic valve with an effective orifice area (EOA) compatible with severe stenosis, a low transvalvular velocity or pressure gradient suggestive of moderate stenosis and a low left ventricular ejection fraction (LVEF).^1^ Dobutamine stress echocardiography (DSE) may aid in the distinction between patients with true severe AS and those with pseudo-severe AS by promoting a potential increase in flow. Hence, traditional hemodynamic indices of stenosis severity could be evaluated at normal flow rates and easily interpreted.^2^ The main limitation of this exam is the unpredictability of flow augmentation, leading to ambiguous changes of mean pressure gradient and EOA.^3^ Projected aortic valve area at normal transvalvular flow rate (250 mL/min) - EOA_proj_ - is an echocardiographic parameter that was developed in order to overcome this limitation. It consists of the effective orifice aortic area that would have occurred at a standardized flow rate of 250 mL/min, enabling the comparison of AS severity between patients with different flow rate profiles with dobutamine infusion.^4^ The determination of this new parameter requires the calculation of at least the basal and peak flow rate in each patient. The original formula of EOA_proj_ published by Blais *et al*. proposed the calculation of flow rate as the quotient between stroke volume and the ejection time (ET), which requires 3 different measurements: 1) left ventricular outflow tract (LVOT) diameter; 2) LVOT velocity-time integral and 3) ET measured at the aortic velocity spectrum.^4^ Flow rate can also be determined by the product of LVOT area and LVOT mean velocity, which requires only 2 measurements: 1) LVOT diameter and 2) LVOT mean velocity.^5^ This alternative method to calculate flow rate is less cumbersome and less susceptible to inter-observer and intra-observer variability as it requires less measurements.

The aim of the present study is to measure the agreement between two methods of calculation of simplified EOA_proj_ using two different approaches of flow rate determination in patients with classical LFLG AS.

## Methods

Retrospective observational single-institution study that included all consecutive patients with LFLG AS with depressed LVEF (definition in accordance with the 2014 AHA/ACC Guidelines for the Management of Valvular Heart Disease^1^) referred for DSE evaluation between September/2011 and November/2015.

Patients admitted to the study had to fulfill all the following criteria: 1) age ≥ 18 years old; 2) EOA ≤ 1.0 cm^2^ or EOA indexed to body surface area ≤ 0.6 cm^2^/m^2^ and maximal transaortic velocity (Vmax) < 4 m/s or mean transaortic gradient (Gmean) < 40 mmHg and 3) LVEF < 50%. Patients with more than mild aortic regurgitation or more than mild mitral regurgitation or stenosis were excluded.

After completing DSE, patients were classified into groups in terms of severity of the stenosis in agreement with the 2014 AHA/ACC Guidelines for the Management of Valvular Heart Disease:

- Patients with true severe LFLG AS: EOA ≤ 1.0 cm^2^ with Vmax ≥ 4 m/s at any flow rate
• Patients who did not fulfill the criteria for true severe LFLG AS having: a) EOA ≤ 1.0 cm^2^ with Vmax < 4m/s (persistent area - gradient mismatch), b) EOA > 1.0 cm^2^ with Vmax ≥ 4 m/s or c) EOA > 1.0 cm^2^ with Vmax < 4 m/s (pseudo-severe AS)

### Echocardiographic assessment

Echocardiographic examination was performed using commercially available equipment (Vivid - 7; General Electric Vingmed, Milwaukee, WI) with a 3.5-MHz transducer.

After the acquisition of the baseline study, a low dose dobutamine infusion protocol was begun at 5 ug/Kg body weight per minute, titrated upward in stages of 5 ug/Kg per minute every 5 minutes up to a maximal dose of 20 ug/Kg per minute. Systemic blood pressure and the 12-lead electrocardiogram were monitored throughout the test. Continuous wave Doppler of the aortic valve velocity spectrum and pulsed-wave Doppler of the LVOT velocity spectrum were recorded at baseline and in the last 2 minutes of each stage of the protocol. LVOT diameter was measured in the basal parasternal long axis view and was assumed to have remained constant during the test protocol.

Raw data was stored digitally and analysis was performed off-line by a single independent operator, using the EchoPac Clinical Workstation Software (General Electric, Vingmed, Milwaukee, WI). For each Doppler measurement, three cycles were averaged, avoiding post-extrasystolic beats. Transaortic gradients were calculated using the simplified Bernoulli equation (*∆*P = 4ν^2^, where *ΔP* is in mmHg and *v* is the aortic velocity in m/s). EOA of the aortic valve was calculated from the continuity equation - EOA = CSA_LVOT_ × (LVOT_VTI_ ÷ Ao_VTI_) -, where *EOA* is in cm^2^, *LVOT_VTI_* is the subaortic velocity -time integral and *Ao_VTI_* is the aortic velocity-time integral both in cm. *CSA_LVOT_* is the cross sectional area (in cm^2^) of the LVOT calculated from the LVOT diameter measured in the parasternal long axis view (d in cm) assuming a circular geometry - *CSA_LVOT_ = π x (d/2)(*^2^. Left ventricular end diastolic and end systolic volumes (LVEDV and LVESV, respectively) and LVEF were assessed by standard 4 chamber and 2 chamber views using the biplane Simpson method. Stroke volume (SV) was calculated from the following equation: = LVOT_VTI_ × CSA_LVOT_, where *SV* is in mL/beat, *LVOT_VTI_* is in cm and *CSA_LVOT_* is in cm^2^. Flow rate (Q) was calculated using 2 different methods:

- a classical method using the formula $Q_{\mathrm{classic}}=1000\times \frac{{\mathrm{LVOT}}_{\mathrm{VTI}}\times {\mathrm{CSA}}_{\mathrm{LVOT}}}{\mathrm{TE}}$, where *Q_classic_* is in mL/sec, *LVOT_VTI_* is in cm, *CSA_LVOT_* is in cm^2^and *ET* is the ejection time in ms measured in the continuous wave Doppler of the aortic valve velocity spectrum.^4^
- an alternative method using the formula Q_alternative_ = CSA_LVOT_ × Vmean_LVOT_ × 100, where *Q_alternative_* is in mL/sec, *CSA_LVOT_* is in cm^2^ and *Vmean_LVOT_* is the mean velocity of blood in the LVOT during the ejection period in m/sec and is measured in the pulsed-wave Doppler of the LVOT velocity spectrum.^5^

Patients with flow rate variation with dobutamine infusion ≥ |15|% in both classical and alternative methods were selected and simplified aortic valve area at 250 mL/s flow rate (EOA_proj_) was calculated according to the formula published by Blais et al^4^: ${\mathrm{EOA}}_{\mathrm{proj}}={\mathrm{EOA}}_{\mathrm{basal}}+\frac{\Delta \;\mathrm{EOA}}{\Delta \;Q}\times \left(250-Q_{\mathrm{basal}}\right)$, where *EOA_proj_* is in cm^2^, *Q* is the mean transvalvular flow rate, *EOA_basal_* and *Q_basal_* are the EOA and Q at rest and *ΔEOA* and *ΔQ* are the absolute variation in EOA and Q with dobutamine infusion.^4^ As we used two different methods to calculate flow rate we obtained two sets of values of simplified EOA_proj_ in each eligible patient: 1) a classical simplified EOA_proj_ using the classical method of flow rate calculation and 2) an alternative simplified EOA_proj_ using the alternative method of flow rate calculation.

### Statistical analysis

Categorical variables are described by frequencies and percentages. Continuous variables are presented as mean ± standard deviation.

A scatter plot and a linear regression model were constructed to assess the strength of linear relation between the classic and the alternative methods of calculation of EOA_proj_ and to quantify the proportion of variance that the two methods have in common. Finally, in order to evaluate the agreement between the two methods (i.e., how much the new method is likely to differ from the old), we built a Bland-Altman plot - a plot of the paired differences between the two methods against their mean. Normal distribution of the paired differences was verified by the use of Shapiro-Wilk normality test. The bias was computed as the mean of the differences of the two methods. A one sample *t* test was conducted against the null hypothesis of no bias to evaluate the statistical significance of the calculated bias. Ninety-five percent-limits of agreement were computed as the mean bias plus or minus 1.96 time its standard deviation.^6^ Two-tailed p values < 0,05 were considered statistically significant.

IBM SPSS Statistics version 23 (IBM, Vienna, Austria) and GraphPad Prism version 7.0 (GraphPad Software, La Jolla California, USA) were used for statistical analysis.

## Results

### Baseline characteristics

Between September/2011 and November/2015, 22 patients [15 (68%) men, mean age 72 ± 9 years] with classical LFLG AS underwent a low dose dobutamine stress echocardiography in order to evaluate the true severity of the AS. No major adverse events were reported. Table 1 shows the baseline clinical and echocardiographic features of these patients as well as the hemodynamic evolution with dobutamine infusion. 8 (36%) patients reached the AHA/ACC criteria for true severe aortic stenosis, 11(50%) patients maintained the valve area - gradient discordance present at baseline and 3 (14%) patients showed a progression of hemodynamic indices suggestive of pseudo severe aortic stenosis. No patient ended up the stress exam with inversion of the area - gradient mismatch (ie, aortic valve area > 1,0 cm^2^ and Vmax ≥ 4 m/s).

**Table 1:** Clinical and echocardiographic characteristics of the low-flow low-gradient aortic stenosis patients at baseline and at 20 ug/Kg/min Dobutamine infusion

**Low Flow Low Gradient Aortic Stenosis (n = 22)**		
**Demographics and Physical Examination**		
Age, yr	72 ± 8.8	
Male sex, n (%)	15 (68)	
Weight, Kg	71 ± 12.7	
Height, cm	163 ± 8.4	
Body surface area, m^2^	1.76 ± 0.183	
**Hemodynamic Indices**		
	**Basal**	**Peak Dobutamine**
Heart rate, bpm	66 ± 8.9	80 ± 18,9
Systolic Blood Pressure, mmHg	115 ± 20.7	139 ± 31,3
Diastolic Blood Pressure, mmHg	62 ± 12.1	64 ± 18,9
Classic Q, mL/s	202 ± 63.3	236 ± 56,3
Alternative Q, mL/s	169 ± 51.2	223 ± 53,9
SV, mL	54 ± 16.0	62 ± 14,4
SVI, mL/m^2^	30 ± 8.4	35 ± 8,7
LVEDV, mL	145 ± 56.9	136 ± 41,7
LVESV, mL	97 ± 42.9	79 ± 38,5
LVEF, %	33 ± 9.8	43 ± 15,3
**Indices of Aortic Stenosis Severity**		
	**Basal**	**Peak Dobutamine**
V_max_, m/s	3.2 ± 0.50	3,9 ± 0,55
G_mean_, mmHg	24 ± 7.3	37 ± 12,2
VTI Ratio	0.22 ± 0.06	0,25 ± 0,07
EOA, cm^2^	0.43 ± 0.091	0,49 ± 0,116
EOAi, cm^2^/m^2^	0.44 (0.35 - 0.50)	0,46 (0,43 - 0,54)
**Classification of Aortic Stenosis in Terms of Severity**		
True Severe Low Flow Low Gradient AS, n (%)	8 (36)	
Pseudo-Severe Low Flow Low Gradient AS, n (%)	3 (14)	
Persistent Area-Gradient Mismatch Low Flow Low Gradient AS, n (%)	11 (50)	
**Simplified Aortic Valve Area at flow rate 250 mL/min**		
Classic EOA_proj_, cm^2^	0.93 ± 0.220 (n = 14)^*^	
Alternative EOA_proj_, cm^2^	0.98 ± 0.238 (n = 14)^**^	

Data are presented as mean ± standard deviation or number (%) of patients, as appropriate. Classic Q: flow rate calculated by the classic formula; Alternative Q: flow rate calculated by the alternative formula; SV: stroke volume; SVI: stroke volume index; LVEDV: left ventricular end diastolic volume; LVESV: left ventricular end systolic volume; LVEF: left ventricular ejection fraction; V_max_: maximum velocity of aortic Doppler spectrum; G_mean_: transaortic mean pressure gradient; VTI Ratio: velocity time integral ratio; EOA: effective orifice aortic valve area; EOA_i_: indexed effective orifice aortic valve area; Classic EOA_proj_: simplified projected aortic valve area calculated using the classic flow rate formula; Alternative EOA_proj_: simplified projected aortic valve area calculated using the alternative flow rate formula; AS: aortic stenosis.

* Only 14 patients had a flow rate variation with dobutamine infusion estimated with the classical formula ≥ |15| %, enabling the calculation of the classic EOA_proj_.

** Only 14 patients had a flow rate variation with dobutamine infusion estimated with the alternative formula ≥ |15| %, enabling the calculation of the alternative EOA_proj_.

Flow rate at baseline and at peak dobutamine infusion was calculated using both the classic $\left(Q_{\mathrm{classic}}=1000\times \frac{{\mathrm{LVOT}}_{\mathrm{VTI}}\times {\mathrm{CSA}}_{\mathrm{LVOT}}}{\mathrm{ET}}\right)$ and the alternative equations (Q_alternative_ = CSA_LVOT_ × Vmean_LVOT_ × 100) in all patients. Only 9 (41%) patients achieved a flow rate variation with dobutamine infusion assessed by both methods ≥ |15|%, enabling the simultaneous determination of the simplified projected aortic valve area at normal flow rate by the classic and the alternative formulas. Table 2 shows the baseline and peak dobutamine echocardiographic characteristics of this subset group of patients.

**Table 2:** Clinical and Echocardiographic Characteristics of the Low Flow Low Gradient Aortic Stenosis Patients with Flow Variation calculated by both methods ≥ |15| % with Dobutamine Infusion

**Low Flow Low Gradient Aortic Stenosis with Classic and Alternative ΔQ ≥ |15|% (n = 9)**		
**Demographics and Physical Examination**		
Age, yr	73 ± 7,1	
Male sex, n (%)	6 (67)	
Weight, Kg	67 ± 13,0	
Height, cm	162 ± 5,8	
Body surface area, m^2^	1,70 ± 0,164	
**Hemodynamic Indices**		
	**Basal**	**Peak Dobutamine**
Heart rate, bpm	67 ± 10,6	81 ± 19,8
Systolic Blood Pressure, mmHg	113 ± 23,9	134 ± 35,2
Diastolic Blood Pressure, mmHg	60 ± 12,6	58 ± 14,1
Classic Q, mL/s	174 ± 45,3	155 ± 42,3
Alternative Q, mL/s	254 ± 55,5	242 ± 56,7
SV, mL	47 ± 13,9	65 ± 15,0
SVI, mL/m^2^	28 ± 6,9	38 ± 8,4
LVEDV, mL	155 ± 74,9	129 ± 46,6
LVESV, mL	107 ± 47,2	72 ± 25,6
LVEF, %	30 ± 9,5	42 ± 13,7
**Indices of Aortic Stenosis Severity**		
	**Basal**	**Peak Dobutamine**
V_max_, m/s	3,2 ± 0,47	4,0 ± 0,64
G_mean_, mmHg	24 ± 5,7	39 ± 13,9
VTI Ratio	0,20 ± 0,056	0,27 ± 0,066
EOA, cm^2^	0,68 ± 0,185	0,94 ± 0,238
EOAi, cm^2^/m^2^	0,40 ± 0,093	0,55 ± 0,126
**Classification of Aortic Stenosis in Terms of Severity**		
True Severe Low Flow Low Gradient AS, n (%)	4 (44)	
Pseudo-Severe Low Flow Low Gradient AS, n (%)	2 (22)	
Persistent Area-Gradient Mismatch Low Flow Low Gradient AS, n (%)	3 (33)	
**Simplified Aortic Valve Area at flow rate 250 mL/min**		
Classic EOA_proj_, cm^2^	0,94 ± 0,246	
Alternative EOA_proj_, cm^2^	0,98 ± 0,248	

Data are presented as mean ± standard deviation or number (%) of patients, as appropriate. ΔQ: variation of flow rate from the baseline with dobutamine infusion, presented as fractional change (%); Classic Q: flow rate calculated by the classic formula; Alternative Q: flow rate calculated by the alternative formula; SV: stroke volume; SVI: stroke volume index; LVEDV: left ventricular end diastolic volume; LVESV: left ventricular end systolic volume; LVEF: left ventricular ejection fraction; V_max_: maximum velocity of aortic Doppler spectrum; G_mean_- transaortic mean pressure gradient; VTI Ratio: velocity time integral ratio; EOA: effective orifice aortic valve area; EOAi: indexed effective orifice aortic valve area; Classic EOA_proj_: simplified projected aortic valve area calculated using the classic flow rate formula; Alternative EOA_proj_: simplified projected aortic valve area calculated using the alternative flow rate formula; AS: aortic stenosis.

**Figure 1:**
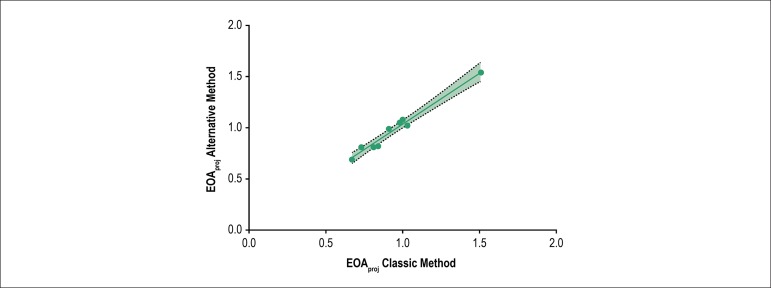
Scatter plot showing the classic simplified projected aortic valve area values against the alternative simplified projected aortic valve area values with a superimposed regression line (solid line) with 95% confidence bands (dashed lines).

A scatter plot showing the classic simplified projected aortic valve area values against the respective alternative simplified projected aortic valve area values was built (Figure 1). As suggested by the scatter plot, a strong linear association between the two methods of calculation was found - *r* (7) = 0,99, p < 0,001.

Simple regression was conducted to find the best line that predicts the simplified projected aortic valve area calculated by the alternative method from the simplified projected aortic valve area calculated by the classic method. The results were statistically significant, F (1,7) = 245,5, p < 0,0001. The identified equation to understand this relationship was: alternative EOA_proj_ = 1.00 (95% CI 0.85 - 1.15) x Classic EOA_proj_ + 0,036 (95% CI -0.111 - 0.182). The adjusted *R*^2^ was 0.97, meaning that 97% of the variance of the alternative EOA_proj_ can be explained by classic EOA_proj_.

**Figure 2:**
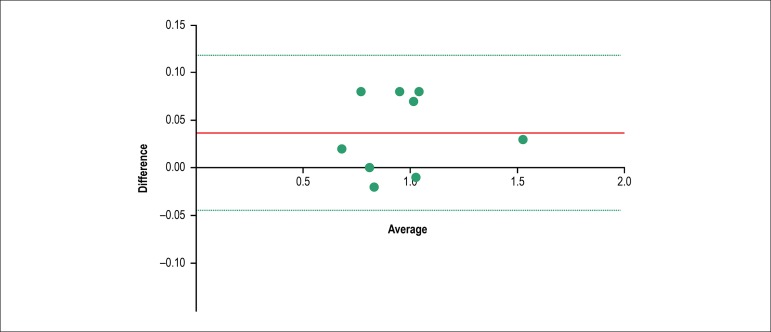
Bland-Altman plot, in which the difference of the two paired EOA_proj_ measurements is plotted against their mean. The solid line parallel to the x axis represents the bias and the dashed lines parallel to the x axis represent the limits of agreement.

A Bland-Altman analysis was performed to assess agreement between the two methods of EOA_proj_ calculation. In Figure 2 the Y axis shows the differences between the two paired EOA_proj_ measurements (alternative method - classic method) and the X axis represents the average of these measurements $\left(\frac{\mathrm{Alternative}\;\mathrm{method}+\mathrm{Classic}\;\mathrm{method}}{2}\right)$. Normal distribution of the differences between paired measurements was verified by use of the Shapiro-Wilk test for normal distribution (test statistics = 0,854, df = 9, p = 0,082). There is no trend in increases in the variability of the differences in relation to their mean. The calculated bias (the average of the paired differences) is 0.037 cm^2^ (95% CI 0.004 - 0.066), meaning that on average EOA_proj_ calculated by the alternative method measures 0.037 cm^2^ more than EOA_proj_ calculated by the classic method. This bias is statistically significant (*t* = 2.619, *df* = 8, p = 0.031). The calculated 95% limits of agreement between the two methods are -0,04 and 0,12, which means that for 95% of the individuals, the EOA_proj_ calculated by the alternative method would be between 0,04 cm^2^ less and 0,12 cm^2^ more than the EOA_proj_ calculated by the classic method.

## Discussion

The EOA_proj_ is defined as the EOA of the aortic valve that would have occurred at a hypothetical standardized flow rate of 250 mL/s. This new echocardiographic index was developed in order to overcome the variable and unpredictable effect of dobutamine in flow rate.^4^ In fact, patients with classic LFLG AS undergoing DSE have a wide variable response in terms of flow rate progression, which may be due to multiple factors including the variable presence of myocardial contractile reserve, the unpredictable chronotropic response to dobutamine and the potential development of left ventricle dyssynchrony with dobutamine infusion^3^ Such variability in flow rate response may impose an insurmountable obstacle in the interpretation of ambiguous changes in mean pressure gradient and EOA. By normalizing the EOA at a hypothetical flow rate of 250 mL/s, the EOA_proj_ enables direct comparison of AS severity in patients with classic LFLG AS that present different flow rate profiles with dobutamine infusion. In addition to make the interpretation of DSE results easier, this new parameter has also been shown to be related to actual AS severity (calcification at surgery) and to have an important value in mortality prediction.^4,7^

In order to calculate the EOA_proj_, EOA is plotted against the mean transvalvular flow rate at different stages of DSE. The slope of this curve - called compliance - is then used to predict EOA at 250 mL/min.^4^ A simplified version of the original formula substitutes the curve slope for an easier to calculate quotient $\frac{\mathrm{Peak}\;\mathrm{EOA}-\mathrm{Rest}\;\mathrm{EOA}}{\mathrm{Peak}\;Q-\mathrm{Rest}\;Q}$. Thus, the simplified version of the EOA_proj_ formula can be expressed as ${\mathrm{EOA}}_{\mathrm{proj}}={\mathrm{EOA}}_{\mathrm{basal}}+\frac{\mathrm{Peak}\;\mathrm{EOA}-\mathrm{Rest}\;\mathrm{EOA}}{\mathrm{Peak}\;Q-\mathrm{Rest}\;Q}\times \left(250-Q_{\mathrm{rest}}\right)$.^8^

Both the original and simplified version of the EOA_proj_ formulae recommend the calculation of flow rate as the quotient between stroke volume and ET which requires 3 different measurements: 1) LVOT diameter (LVOT_D_); 2) LVOT velocity-time integral (LVOT_VTI_) and 3) ET measured at the aortic velocity spectrum. Both LVOT_D_ and LVOT_VTI_ are measures routinely done in DSE protocols performed for classic LFLG AS evaluation as they are needed to calculate EOA of the aortic valve by the continuity equation. However, the need for ET measured at the aortic velocity spectrum adds the requirement for an extra measurement in the usual protocol of DSE. Furthermore, this flow rate formula involves measurements acquired in different places and, inevitably, in different time points, encompassing an intrinsic bias.

Flow rate can also be determined by the product of left ventricular outflow tract area and left ventricular outflow tract mean velocity, which requires only 2 measurements: 1) LVOT_D_ and 2) mean velocity of blood at LVOT during the ejection period (LVOT_Vmean_). LVOT_Vmean_ is given automatically in most echocardiography software when assessing LVOT_VTI_ (a fundamental step in EOA calculation by the continuity equation). This alternative formula is less cumbersome to calculate as it does not need an additional measurement in the aortic velocity spectrum. Also, as it only requires 2 different measurements, it is less prone to increased inter-observer and intra-observer variability.

This study aimed to assess how much the EOA_proj_ calculated using an alternative method to estimate flow rate differs from the EOA_proj_ calculated by the standard formula. The Bland-Altman method was used to assess agreement between the two methods. As previously published, Pearson correlation and linear regression analysis can be misleading in terms of assessing agreement between two measurement methods, as data which seem to be in poor agreement (for instance, a change in scale of measurement) can be highly correlated.^6,9^ Bland-Altman method assesses how well the methods agree on average (by estimating the mean of the differences for individuals - the systematic bias) and how well the measurements agree for individuals (by examining the variability of the differences and the calculation of the limits of agreement which quantify the range of values that can be expected to cover agreement for most of the subjects).^10^

Using the Bland-Altman method, we found a systematic bias of 0.037 cm^2^ (95% CI 0.004 - 0.066), meaning that on average the alternative method overestimates the EOA_proj_ in 0,037 cm^2^ compared to the classic method. Despite being statistically significant, this bias is not clinically significant as it is less than 0.1 cm^2^. Also, the 95% limits of agreement are quite narrow (from -0,04 cm^2^ to 0,12 cm^2)^, meaning that for 95% of individuals, EOA_proj_ calculated by the alternative method would be between 0,04 cm^2^ less to 0,12 cm^2^ more than the EOA_proj_ calculated by the classic equation. Such narrow range is the largest likely differences between the two methods, and do not compromise the clinical agreement between the two methods. Therefore, it is reasonable to acknowledge the potential interchangeability of the two methods of EOA_proj_ calculation in clinical practice.

## Conclusion

This study presented a new method to calculate the simplified EOA of the aortic valve at normal flow rate using a less cumbersome equation to estimate flow rate and tested the agreement of this new method with the previous reported by Blais et al.^4^ The bias and 95% limits of agreement of the new method are narrow and not clinically relevant, supporting the potential interchangeable use of both methods in clinical practice. As the new method requires less additional measurements, it would be easier to implement it in clinical practice, promoting an increase in the use of EOA_proj_ - a valuable echocardiographic parameter in the evaluation of LFLG AS.

### Limitations

This is a small retrospective single-institution study that is inherently underpowered to assess small differences in echocardiographic variables between groups. A higher number of patients is needed to investigate potential discrepancies in the performance of both EOA_proj_ calculation methods in different subsets of LFLG AS patients. Therefore, the results presented here must be interpreted with caution.
